# The magnitude and direction of the relationship between risk factor and cognition depends on age: a pooled analysis of 5 community-based studies

**DOI:** 10.1007/s10654-023-01087-0

**Published:** 2024-01-05

**Authors:** Osorio Meirelles, Anthony Arnette, Vilmundur Guðnason, Lenore J. Launer

**Affiliations:** 1 Laboratory of Epidemiology and Population Sciences, Intramural Research Program, National Institute on Aging, 251 Bayview Blvd., Baltimore, MD 21224 USA; 2 Icelandic Heart Association, Kopavagur, Iceland; 3 University of Iceland, Reykjavik, Iceland

**Keywords:** Cognitive impairment, Cardiovascular risk factor, Age modification of risk, Life-course

## Abstract

The mixed evidence of the association between high levels of cardiovascular risk factors (CVRF) and the risk for cognitive impairment may be due to confounding of age across studies. We pooled and harmonized individual-level data (30,967 persons, age range 42–96 years) from five prospective cohorts to investigate by 1 year age increments to investigate whether or not there is change in slope describing the association of CVRF to a cognitive outcome (Digit Symbol Substitution Test; DSST). The CVRF included: systolic and diastolic blood pressure, total cholesterol, fasting glucose and body mass index. Linear and quadratic piecewise regression models were fit to the trajectory patterns of these slopes (betas). The pattern of yearly slope changes showed higher CVRF were associated with lower DSST, but associations attenuated toward zero as age increased for all but DBP where 1 year slopes for DBP changed direction from negative to positive from mid- to late-age. Age is not only a driver of cognitive decline—age also modifies the direction and strength of the association of cognitive function to CVRF and cohort age may be one reason why the evidence for CVRF-CD association is mixed.

**Supplementary Information:**

The online version contains supplementary material available at 10.1007/s10654-023-01087-0.

## Introduction

Late-life cognitive impairment and dementia (CD) are devastating. The evolution of these conditions begins long before clinical onset so early prevention interventions are needed [1]. Because of their potential for modification and the availability of data from life-course epidemiologic studies, control of cardiovascular risk factors (CVRF) has been targeted as a class of risk factors that could be intervened on to prevent or slow the progression of CD. However, observational and trial evidence of the magnitude of the association between CVRF and CD is mixed, and it is still unclear which CVRF during which age period would be the most effective approach to reduce the burden of CD [2]. This gap has slowed the development of effective interventions on CVRF aimed to reduce the burden of CD.

Evidence of the mixed findings is in the variation in the slopes, risk ratios or significance of the relationship between a CVRF (i.e., blood pressure) and CD outcomes. Reports from observational studies range from an expected higher CVRF level increasing the risk for CD, to a null association, to an unexpected lower CVRF level increasing the risk for CD [3]. The few clinical trials that test the effects on cognitive impairment of intervening on a single cardiovascular risk factor have largely been negative [4–6] with the exception of SPRINT MIND [7].

Reconciling these mixed findings is hampered because they are based on single studies of different aged cohorts (i.e. ≤ 55; ≥ 55, ≥ 65 or ≥ 75 years. of age), different study designs (i.e. cross-sectional, case–control or longitudinal), different target populations (i.e. clinical volunteers, patient groups, or population-based), different outcome measures (i.e. cognitive testing, clinical diagnosis, or algorithmic classification) and follow-up time as well as different statistical models to test for associations. Across cohorts, there may be a different balance of confounding variables, which could bias not only the internal validity of the study, but also the validity of cross-cohort comparisons. It is also a challenge to study early risk factors because CD is, by definition, a condition of late-life with initially slow progression [8].

Age of an individual or a study cohort is a core feature that can account for the inconsistencies in the literature. Here, we are interested in the extent to which, and the pattern of how, age modifies the magnitude and direction of the association between CD and CVRF. This question differs from those that include age as an independent correlate, as a confounder, or as a scale to show longitudinal trajectories of a variable. Testing for this age interaction is similar to studies presenting age-stratified analysis to account for possible differences in the association of a CVRF and cognitive outcome by age strata [2, 3]. However, such age-stratified analyses often include a wide age range and assume a constant association between the risk factor (RF) and outcome over the age interval. Further, the studies of mid-life CVRF to late-life cognition can have up to a 15–20 years gap between measures, so there are few data giving insight into the evolution of change in the association. Understanding this evolution can help design future prevention and precision medicine approaches and also contribute to our interpretation of observational studies.

Here we aim to test the assumption of a constant association of RF to CD over a wide age range. We calculated 1 year slopes of these associations for five CVRF and modeled the trajectory of these 1 year age slopes from mid-to late-life. These 1 year slopes are derived from pooled individual-level data from five mid- and late-life community-based cohorts that have measured the same cognitive test and set of CVRFs. We modelled the trajectory of these 1 year slopes to investigate whether there is a negative association between CVRF and CD throughout mid- to late life or whether these associations change over time.

## Methods

### Cohort descriptions and variable selection

*Cohorts*. The following five cohorts of men and women were included in our synthetic cohort: Age Gene/Environment Susceptibility-Reykjavik Study (AGES-RS; baseline 2002/06, n = 5764, 67–96 years, all European Caucasian [9]; Atherosclerosis Risk in Communities study (ARIC, 1987/89, baseline n = 15,792, 44 to 64 years, bi-racial [10]; Cardiovascular Health Study (CHS, baseline 1989, n = 5888, 65–102 years, bi-racial [11]; Coronary Artery Disease in Young Adults study (CARDIA, baseline 1984/85, n = 5115, 18–30 years, bi-racial [12]; and the Multi-ethnic Study of Atherosclerosis (MESA, baseline 2000/02, n = 6814, 44–84, 4 race/ethnicity groups [13]. The cohorts are briefly described in Online Resource 1. The first cognitive tests in MESA, were acquired at the 5th exam; CARDIA at the 25 years follow-up exam; AGES-RS at the baseline exam; ARIC at the 2nd follow-up exam; and CHS at the baseline exam (See Online resource 2 which gives the mean age and calendar years of data collection for the exams that were included in this analysis).

*Risk factors*. Five cardiovascular risk factors were selected for study: diastolic (DBP) and systolic (SBP) blood pressure (mmHG), body mass index (BMI, kg/m^2^), total cholesterol (mg/dL) and glucose (mg/dL). These risk factors have an established body of research into their association with CD and have been robustly measured in all cohorts using standardized methods [2, 3, 14–17].

#### Cognitive outcome

As dementia occurs only in later years with no events in middle age, we chose to study a cognitive outcome and chose the Digit Symbol Substitution Test (DSST) [18] because it was measured in all 5 cohorts, is informative in mid and late-life [12, 14] and predicts future, or reflects, current dementia [19, 20]. The DSST is an omnibus test of processing speed, visuospatial skills, and sustained attention and has an approximately normal distribution in mid and late-life individuals. The DSST is a standard paper-and-pencil based timed-test where the participant writes down symbols paired with a digit (133 pairings), as presented in a set of pairs given at the top of the page. However, time to complete the pairings differed by study (ARIC and CHS allowed participants 90 sec for the test, and AGES-RS, CARDIA, and MESA allowed 120 sec) thus changing the achievable maximum score. Additionally, the distribution and range of the DSST scores are influenced by cohort differences in age, sex, race/ethnicity and education of the participants [3, 10, 21]. Therefore, we generated a harmonized DSST score that was comparable across cohorts (see below).

#### Covariates

Because of their strong association with CVRF and DSST [10], the harmonization models included baseline age, sex, race/ethnicity (White vs. Other), education (< high school, high school, > high school) and smoking status (never smoked, past smoker, current smoker).

#### Analytical sample

The harmonization sample was restricted to those with, per exam, complete data on DSST and CVRF, as shown in Online resource 2, which includes a figure describing the study design and the exams included in the current analysis. After filtering, the analytical sample included: AGES-RS, n = 5342, age range 66–96 years; ARIC, n = 13,698, 46–75 years; CARDIA, n = 3334, 43–59 years; and MESA, n = 4059, 53–94 years, giving an analytical pooled sample of 30,967 persons with an age range of 42–96 yo (see Online resource 3 for the sample size per 1 year age bin).

### Statistical methods

#### Data harmonization

CVRF and the DSST scores were harmonized to the MESA cohort as it had the widest age interval. The harmonization algorithm is described in detail in Online Resource 4 and graphed in Online resource 5. The algorithm is based on previously published methods that harmonize data based on the standard deviation of residuals [22]. For example**,** for one risk factor (DBP) and one cohort (ARIC), the harmonized value of DBP for individual I and timepoint j was estimated as follows: First, a linear regression model ‘DBP = age, sex, education and smoking’ (as defined above) was estimated and the residuals (R_ijARIC_) from that model were generated. These residuals reflect how much the individual differed from the cohort model prediction. Next, based on the MESA data, we ran a linear regression where DBP is predicted by covariates. Then we weighted the ARIC (and the other cohorts separately) covariates with the covariate betas generated from the MESA model, giving an ARIC/MESA-based prediction of DBP. The residuals from the ARIC (R_ijARIC_) model were then added to the predicted ARIC-MESA DBP giving a harmonized DBP data point for ARIC. According to this method, ‘harmonized’ CVRF or DSST data points from MESA are the original MESA. Our method of harmonization is supported by the consistency of the cohort-specific non-harmonized (i.e. original data) compared to harmonized trajectories, as can be seen in the following supplemental materials: Online resource 6a for each cohort non-harmonized compared to harmonized mean values of per 1 year age bin; and Online resource 6b for risk factor-specific, non-harmonized and harmonized plots of combined cohort data on the association of the risk factor to 1 year mean age bins.

In exploratory analyses of each cohort separately, we also compared statistical fit of the main model to one that included additional covariates (i.e., for the DBP model we added fasting glucose, BMI and total cholesterol) (Online resource 7). Comparisons of the R2 and mean square error of models with and without additional confounders did not show meaningful improvement to the model. We did not control for RF-control medications, as we were interested in the association of the DSST to the level of the RF, which captures the effect of medications. Additionally, we stratified models by sex and race (for 4 cohorts) to assess whether stratifying the harmonization models by these demographic c*haracteristics* improved the model, but our tests of interaction of (race or sex)*CVRF were not significant (Online Resource 8a, 8b and 9).

#### Statistical models

To estimate the trajectory of the slope *(T-slope),* data points (i.e. 1 year slopes) from all harmonized cohorts were combined and each individual timepoint was binned by 1 year age groups. To optimize sample size per age bin we combined the 1 year slopes at the lower and upper age range (i.e. age bins ≤ 47 and ≥ 88), giving 42 age bins each with corresponding CVRF-DSST 1 year slope estimate.

The overall pattern of CVRF-DSST 1 year slopes from middle to late age, the slope trajectory (T-Slope), was calculated by modeling the CVRF-DSST 1 year-slope (Y) as a linear function of the age in each age bin (X). Upon inspection of graphical data and confirmed by the poor fit of the linear model [model 1] it was evident that, with age, some 1 year slopes began to increase or decrease for some CVRF-DSST slopes. This suggests the association between a CVRF and DSST changes with age, as described above. Based on the patterns of the trajectories of slopes, we sought to define the best-fitting model. To do this we compared linear, and piecewise linear and quadratic models using the Bayesian information criterion (BIC; MR0468014) (see Online resource 10 for details of model fit).

The linear–linear piecewise model estimated each of the two *T-slopes* (i.e., pieces) as a linear function. The second piecewise model, linear-quadratic, estimated one piece as a linear function, and the second piece as a quadratic function. To decide where to cut the model into two pieces, we estimated *agecut,* the age at which BIC was the lowest, generally resulting in a strong shift in the direction of the *T-slope* (see model details in the Online Resources). For all three models, each data point (CVRF-DSST 1 year-slopes per corresponding age bin) was weighted by the inverse of the square of the standard error of the CVRF-DSST 1 year-slope (See Online Resource 11 for details on the model development).

There is some dependency between age bins since some individuals in a 1 year age bin can also be in another older 1 year age bin. The dependency does not affect the beta, but the standard deviation is estimated to be larger than had it been based on independent observations. This results in a smaller ratio between the beta and standard error leading to an overly optimistic p-value. Bootstrapping is the standard approach to correct for such overly optimistic *p* values [23]. Therefore, to correct for age-bin dependency, we performed 1000 iterations using sampling with replacement (bootstrap) on our initial dataset. For each iteration, we saved the piecewise model parameter estimates to estimate the standard error of those estimates to generate a correct z-score and adjust the p*-*values and confidence intervals accordingly.

We verified the model fit by visually comparing the trajectory estimated by the model to a smoothed trajectory based on a procedure with a triangular smoothing window of size 11 (Online Resource 12 describes the smoothing algorithms) and that accounts for the variance of the 1 year-slopes and number of cases per age bin. Finally, we visually verified the fidelity of the models in each cohort/risk factor combination.

## Results

**Figures 1:**
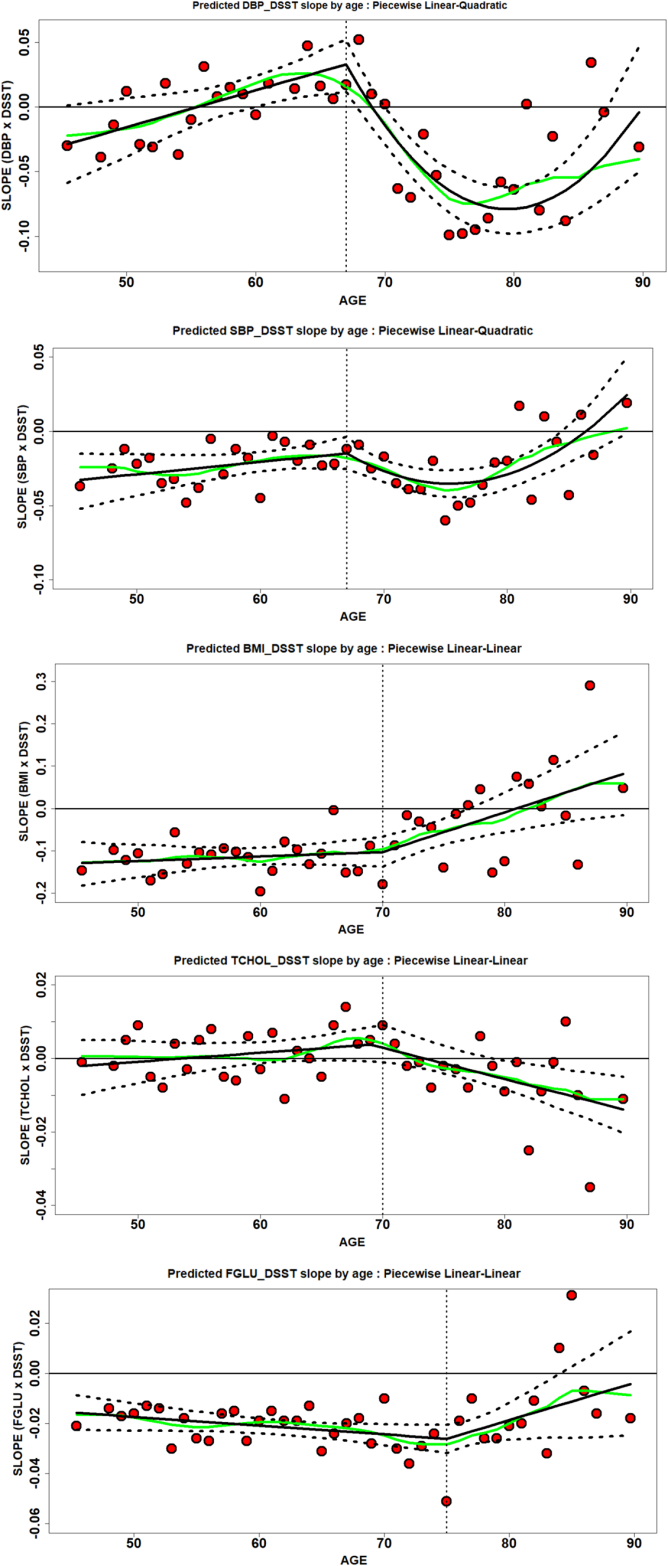
**a**–**e** Five Cardiovascular risk factor—DSST relationships by 1 year age bins. Each dot (in red) represents the magnitude and direction of the 1 year association between the risk factor and the DSST cognitive score. The horizonal line at the y-axis zero is the point above which yearly slopes increase and below which decreases. The vertical stippled represents the age in which the first part of the piecewise model end and the beginning of the second part (i.e. Figure for BMI the line is at age 70 years). Green line is the smoothed trajectory; black line is the modeled trajectory and stippled black line is the 95% confidence around the modeled trajectory. Specific risk factors as modeled (2 spline components): 1a. DBP—linear-quadratic; 2a. SBP—linear-quadratic; 3a. BMI—linear–linear; 4a. cholesterol—linear–linear; 5a. Fasting glucose—linear–linear. See model parameters in the text. parameters in the text

The percent women was generally similar across cohorts (range 42.1–46.8%). At baseline (Table 1), ARIC and CARDIA had the youngest participants and AGES-RS the oldest. Trends in CVRF and DSST across cohorts generally tracked with the relative ages of the cohorts. The mean and standard deviation of harmonized and unharmonized variables are shown in Online Resource 13. The mean of the observed DSST-CVRF 1 year slope per age bin, the modelled trajectory of the 1 year slopes, the smoothed trajectory of the 1 year slope, and 95% CI of the trajectory are shown in Fig. 1a–e. The predicted and smoothed trajectories by study and by CVRF are shown in Online Resource 14.

**Table 1:** Description of all participants with harmonized and complete data in pooled analysis by cohort

	AGES-RS	ARIC	CARDIA	CHS	MESA
N persons	5342	13,698	3334	4534	4059
N observations	8299	26,028	3334	6853	4059
Age y (mean/range)	77.7 (66–96)	59.8 (46–75)	50.2 (42–59)	74.4 (63–95)	69.4 (53–94)
Sex (% females)	42.3	44.5	43.6	42.1	46.8
Diastolic BP (mmHg)^a^	65.4 (10.3)	69.4 (10.3)	71.8 (10.9)	66.1 (12.8)	68.5 (10.1)
Systolic BP (mmHg)^a^	126.4 (20.7)	116.9 (18.6)	111.6 (15.3)	124.4 (21.7)	123.8 (20.7)
Body mass index^a^	27.3 (4.2)	29.2 (5.4)	29.6 (7.0)	27.6 (4.2)	28.4 (5.6)
Total Cholesterol^a^ (mg/dL)	178.2 (44.3)	187.8 (39.9)	195.5 (37.9)	180.6 (39.2)	183.3 (37.1)
Fasting Glucose^a^ (mg/dL)	98.8 (21.2)	101.5 (40.6)	101.4 (28.5)	98.9 (31.9)	102.0 (28.7)
DSST^a^ (no. correct)	45.3 (12.3)	58.4 (14.1)	69.7 (15.3)	48.4 (14.0)	50.6 (18.3)
Smoking (%current)	10.8	19	18.7	7.8	7.8
Education (% < 12 years.)	19.6	20.3	1.7	23.7	14
White (%)^b^	100	76.7	53.7	95.8	40.4

*CHS* the cardiovascular health study, *ARIC* atherosclerosis risk in the communities study, *MESA* the multi-ethnic study of atherosclerosis, *CARDIA* the coronary artery risk development in young adults, *AGES-RS* the age gene/environment susceptibility-reykjavik study, *DSST* digit symbol substitution test

^a^Mean (SD)

^b^White compared to Non-white participants

For DBP, the linear-quadratic model (Fig. 1a) had the lowest BIC, with *agecut* = 67. Over the age span, there was considerable variation in the 1 year slopes. For younger ages (less than 55 y) and older ages (≥ 67 year) 1 year slopes were mostly negative (i.e., < 0), suggesting higher DBP is associated with lower DSST scores. Between 55 and 70 year, 1 year slopes attenuated and became positive close to zero (p = 5.3E−3). Although still positive at age 67, after this, slopes start a downward trend and become increasingly negative until approximately 80 years of age, when again the 1 year slopes increasingly attenuate to zero (*Tslope2sq* = 0.00072 (0.00045,0.00099; *p* = 2.4E−7). This translates into an estimated 0.013 higher DSST score at age 60 years; a 0.014 lower DSST score at age 70 years; and a 0.079 lower DSST score at age 80 years for each increment in 1 mmHg DBP, controlling for covariates.

Similar to DBP, the SBP (Fig. 1b) linear-quadratic model had the lowest BIC, with *agecut* = 67 years. Individual 1 year SBP-DSST-slopes were negative across the age span but became less negative as age reached 67 years and then became more negative as age reached the relative minimum (nadir), of 75.4 years. After age 75 years, the 1 year slopes attenuated with age. Together these changes reflect a U-shaped relationship from age 67 to 88+ years old (T-slope2sq = 0.00029 (0.00014,0.00044; *p* = 1.3E−4). Based on this model, with each increment in 1 mmHg SBP, adjusting for covariates, there is an estimated 0.021 lower DSST score at age 60 yo; a 0.027 lower DSST at age 70 yo; and at age 80 yo there is a 0.029 lower DSST score.

The BMI trajectory was best modeled (lowest BIC) by the linear–linear model (Fig. 1c) analysis, with *agecut* = 70 years. For age ≤ 70, for each unit increase in the 1 year slopes of DSST-BMI, there was an average 0.12 lower DSST; however, the trajectory is not significant (p = 0.5) and approximately constant. After age 70 years, 1 year-slopes become increasingly less negative with age (p = 1.5E-3), attenuating to zero and becoming positive slopes around age 80 years, i.e., a higher BMI was associated with a higher DSST score after 80 years of age. For each increment of 1 BMI unit, this translates to an estimated 0.11 lower DSST score at age 60 years; a 0.10 lower DSST score at age 70 years; and a 0.009 lower DSST score at age 80 years.

The best fitting model for total cholesterol was the linear–linear model (Fig. 1d) and *agecut* = 69. Overall, 1 year slopes were constant and close to zero for age < 69 years (p = 0.29). After age 69 years, the magnitude of the 1 year-slopes of the relationship between DSST and cholesterol tended towards more negative such that a higher cholesterol was associated with a lower DSST score (p = 2.2E-3). This translates to an estimated 0.0016 higher DSST score with each increment in 1 mg/dL of cholesterol at age 60 years; a 0.0029 higher DSST score at age 70 years; and 0.0056 lower DSST score at age 80 years.

The linear–linear model for fasting glucose (Fig. 1e), had the lowest BIC, with *agecut* = 75 years. Overall, 1 year slopes for DSST-fasting glucose associations were negative, meaning that for a unit increase in fasting glucose, there was a lower DSST score. The linear trends before (*p* = 0.07) and after (*p* = 0.057) age 75 were relatively constant but attenuate to zero. For age ≤ 75 years, the expected 1 year-slopes were marginally lower by 0.00035 for each increase in 1-unit of fasting glucose. After age 75 years, the expected 1 year-slopes was 0.0015 points higher with each 1-unit increment of age, with a drift of the 1 year slopes attenuating towards zero. This translates into an estimated 0.021 lower DSST score at age 60 years; a 0.024 lower DSST score at age 70 years; and a 0.019 lower DSST score at age 80 years for each increment in 1 unit of fasting glucose level.

## Discussion

In 2018, the National Academy of Science summit on prevention of cognitive decline [Preventing Cognitive Decline and Dementia: A Way Forward, National Academies Press, http://www.nap.edu; and https://www.nia.nih.gov/research/administration/recommendations-nih-ad-research-summit-2018] concluded the evidence for control of CVRF to reduce the risk for CD is not robust or consistent enough to support prevention trials or for public health messaging. The design of effective pharmacological or behavioral approaches continues to be priority and to move forward, more insight is needed on factors underlying the incongruent results.

We found, controlling for study design and methodology (including only prospective community-based cohorts, standardized measurements of CVRF and cognition, harmonized variables, and adjusting for major confounders of sex, race, education and smoking), the five CBRF we studied were *negatively* associated with DSST test scores in both middle- and late-age. However, the magnitude and direction of the CVRF-DSST slope changed with age, such that as age increased, the association of CVRF to DSST attenuated towards zero suggesting no association between the two factors. There were CVRF differences in the age at which the magnitude or direction of slopes was estimated to change, but generally slopes began to differ between the late 60 s and mid 70 s age-band. BP, and specifically DBP showed the most variable 1 year changes in the slope of its association to DSST. Based on the differences in DSST SD units per one unit increase in RF, at age 60 years the DSST scores were lowest for BMI, at 70 years SBP, and at 80 years DBP, had the greatest negative effects.

Although this analysis does not address the specific reasons for the direction and magnitude of changes in the slope trajectories, several contributing factors to the age-related loss of the CVRF explanatory or predictive power can be proposed. First, as a result of the increase in multi-morbidity with aging, there may be a proportional reduction in how much any *one* risk factor can explain CD. An oft suggested explanation is ‘reverse causality,’ where one hypothesizes that X ‘causes’ Y, but in reality, Y actually ‘causes X.’ For instance, we find high blood pressure is associated with better, not worse, cognitive decline or we find lower weight and cholesterol levels associate with an increased, not decreased, risk for dementia [24, 25]. This reverse causation could reflect underlying biologic, behavioral or methodologic issues [26, 27]. In the case of dementia, it is reasonable to assume behavioral changes, such as loss of appetite or more sedentary behavior, may affect biologic changes resulting in CVRF levels that no longer reflect the past cumulative exposure to the risk factor. Biological changes can result in reverse causality if there is a shift in the balance between the central and peripheral regulation of CVRF, whereby the pathology in the brain regulates the risk factor, and not vice versa. For instance, neurodegenerative pathology in the hypothalamus may affect regulation of glucose, insulin or body weight [28], leading to prodromal changes such as more rapid weight loss and lowering of blood pressure and cholesterol levels as compared to those who maintain cognitive function [24, 29]. Although data are limited on the empirical significance biological changes, epidemiologic and statistical studies suggest failure to account for possible reverse causation can lead to biased conclusions [30].

The variability in the BP trajectories, particularly the DBP during the 70 to 80 years old band, is striking and significant (the quadratic term in the piecewise modeling of the dip is statistically significant). Although standardized methods were used and we harmonized the BP measures across cohorts, blood pressure measures per se are very variable, partly reflecting measurement issues for SBP and DBP, with DBP having added sources of reading error when sphygmomanometers [31] are used. Additionally, at older ages there are many factors that can influence participation and functional performance. With age, SBP tends to increase, while DBP may decrease due to vascular disease such as increased arterial stiffness [32]. Further, compared to the other risk factors we examined, high BP is a more direct measure of mortality risk, so selection bias may be relatively stronger for those with unfavorable BP levels [33, 34] compared so the other CVRF. As result of all these factors, the sample composition in the bracket between 70 and 80 of age could be very heterogeneous across and within studies and this may be reflected in the variability of the trajectory of 1 year slopes.

Our novel approach uses the trajectory of slopes based on pooled population-based cohorts to frame issues that are important to designing future research. [35] Our study points to candidate CVRF, estimates age intervals when the impact of an intervention may be more or least effective, and provides some insight into how long an intervention, how large a sample size, or how big of a treatment effect is needed to achieve a detectable effect. For example, if the CVRF-Cognition association is strongly negative during a particular age interval, then an intervention either before or during the age interval may be more likely to positively change the outcome than during an age period where there is no association. Our study also suggests the effect sizes of any trial are likely to be small so clinical trials conducted at later ages will need large sample sizes or target vulnerable sub-groups and will need to deliver an intervention that creates large differences between treatment arms, such as SPRINT MIND, where the difference in SBP in the treatment versus control arms was > 14 mmHG [36]. Further, the attenuation of the relationships suggests the contributions to poor cognitive function of other risk factors is increasing; this multi-morbidity should be accounted for in patient selection and should be reflected in the extrapolation of the results to more complex populations. Finally, this research can also inform the comparison and integration of findings from the increasing number of observational studies reporting on the CVRF-CD associations. Importantly, if the association varies depending on the age of the cohort, it is reasonable to further investigate any methodologic reasons for the variation before abandoning a candidate RF or before planning an intervention for the RF.

Our analysis has several strengths. By including studies with similar study designs, standardizing the interval between the CVRF and DSST, no imputation of cognitive scores, and applying similar analytical approaches to all the studies, we have minimized these sources of variability present in cross-study comparisons. We also allowed for non-linear 1 year slope trajectories and used BIC criteria to select the best fitting models so we could better capture the patterns of change in slopes over time.

However, several aspects of our study should be noted when interpreting the results. The trajectories are based on cross-sectional binned 1 year slopes of CVRF–DSST associations so the analyses are subject to the same biases as other cross-sectional studies of ‘change by age’—most importantly the bias caused by selective dropout by age and health condition. As a result, there is the possibility that the sample is getting ‘healthier’ with age and this may explain some of the attenuated associations of CVRF to DSST. We used a single cognitive test with a normal distribution of test scores reflecting psychomotor speed and executive function; tests with different properties may give different results. It is also possible that over the 30 years spanned by these 5 cohorts there were secular trends in the CVRF that may affect the magnitude of the associations, but because the cohorts did not perform cognitive testing at all exams, we could not test this [37]. However, a recent article [38] reporting on a NHANES analysis of serial cross-sectional exams from 1999 to 2018 showed there were only ‘sub-optimal’ improvements over time in CV health. Finally, we incorporated relatively few confounding variables in our models, including medications to lower the RF, but our analysis showed including more confounders the model fit did not improve. Never-the-less, we cannot exclude the presence of residual confounding.

In conclusion, understanding the methodologic and demographic differences among observational studies will aid in the interpretation of CVRF—CD associations and promote more targeted designs of clinical trials to prevent cognitive loss in late-life.

### Supplementary Information

Below is the link to the electronic supplementary material.

Supplementary file 1 (PDF 111 KB)

Supplementary file 2 (PDF 2590 KB)

## Data Availability

Data from ARIC, CARDIA, CHS and MESA, were accessed from the NHLBI Biologic Specimen and Data Repository Information Coordinating Center [https://biolincc.nhlbi.nih.gov/studies]. Data from the AGES-RS study are available at the Icelandic Heart Association upon request [https://hjarta.is/en/]. The derived data generated in this research will be shared on reasonable request to the corresponding author (launerl@nia.nih.gov).
