# Supplementary material for: The magnitude and direction of the relationship between risk factor and cognition depends on age: a pooled analysis of 5 community-based studies
Source: Eur J Epidemiol. 2024 Jan 5;39(2):161–9. doi: 10.1007/s10654-023-01087-0 (PMC10904440; doi:10.1007/s10654-023-01087-0)
Supplement: Supplementary file 1 — (PDF 111 KB) [file 10654_2023_1087_MOESM1_ESM.pdf]

Meirelles O[1], Arnette A[1], Gudnason V[2], Launer LJ[1]. The magnitude and direction of the relationship between risk factor and cognition depends on age: a pooled analysis of 5 community-based studies **European J Epidemiology.**

1. Laboratory of Epidemiology and Population Sciences, Intramural Research Program, National Institute on Aging, 251 Bayview Blvd., Baltimore, MD USA 21224
2. Icelandic Heart Association, Kopavagur; University of Iceland, Reykjavik, Iceland

List and legends for Online Resources (OR)

Titles

OR 1: Description of the 5 cohorts included in the pooled analysis

OR 2: Online Resource 2: Mean age (years) and calendar year by exam per cohort

OR 3: Graphed sample size per 1-yr age bin - all cohorts combined cohorts

OR 4: Online Resource 4 Method to harmonize risk factors and DSST across 5 cohorts in the pooled analysis.

OR 5: Graphed steps to harmonize original cohort data to the MESA study

OR 6a: Comparison of non-harmonized and harmonized 1-yr means of CVRF by cohort
OR 6b:Comparison of non-harmonized and harmonized risk factor by age by cohort

OR 7: Comparison of DSST-DBP 1-yr slope trajectories model-fit statistics without (R0) and with (R1) additional confounders (BMI, Fasting Glucose and Systolic BP)

OR 8a: Trajectories of the association of the DSST to CVRF by race.
OR 8b: Trajectories of the association of the DSST to CVRF by sex.

OR 9: Resource : Model fit differences (R2)comparing stratified harmonized models (Race: White and Black; Sex: Men and Women) to pooled sample model (Figure 1a-e, main text)

OR 10: Comparison of statistical fit for different piecewise models estimating 1-yr slope trajectories of the association between a cardiovascular risk factor and the DSST cognitive test outcome.

OR 11: Description of the model development to estimate trajectories of 1-yr slopes describing the relationship between a cardiovascular risk factor and the DSST

OR12: Description of the smoothing algorithm applied to the trajectories of 1-yr slopes shown in Figures 1a-e (main text)

OR13: Table: Cross cohort harmonized and non-harmonized mean and standard deviation of risk factors and DSST cognitive score

OR 14: Trajectories of harmonized 1-yr slopes of the association between the DSST and cardiovascular risk factors by cohort.
