# Supplementary material for: The magnitude and direction of the relationship between risk factor and cognition depends on age: a pooled analysis of 5 community-based studies
Source: Eur J Epidemiol. 2024 Jan 5;39(2):161–9. doi: 10.1007/s10654-023-01087-0 (PMC10904440; doi:10.1007/s10654-023-01087-0)
Supplement: Supplementary file 2 — (PDF 2590 KB) [file 10654_2023_1087_MOESM2_ESM.pdf]

The magnitude and direction of the relationship between risk factor and cognition depends on age:a pooled analysis of 5 community-based studies

European J Epidemiology.

1. Laboratory of Epidemiology and Population Sciences, Intramural Research Program, National Institute on Aging,

251 Bayview Blvd., Baltimore, MD, USA 21224

2. Icelandic Heart Association, Kopavagur; University of Iceland, Reykjavik, Iceland

Corresponding author: LJ Launer, email: launerl@nia.nih.gov

Online Resource 1: Description of the 5 cohorts included in the pooled analysis

Age Gene/Environment Susceptibility- Reykjavik Study (AGES-RS) is a single center prospective study based on the Reykjavik Study, which was initiated in 1967 by the Icelandic Heart Association to study cardiovascular disease and risk factors. A follow-up of randomly selected surviving cohort members was initiated as the AGES-RS study between 2002 and 2006 (n=5764 participants, 42% male), and re-examined in 2007-2011 (n=3316).. Participants were evaluated with a questionnaire and a clinical exam, had a fasting sample of blood drawn, and underwent various bio-imaging measures. The Digit Symbol Substitution Test (DSST) was administered to all participants (https://hjarta.is/en/research/).

The Atherosclerosis Risk in the Community (ARIC) study initially comprised 15,792 participants aged 45-64 years in 1987-1989, recruited from four communities: ARIC performed four examinations of the cohort between 1987 and 1998, followed the cohort primarily with annual telephone interviews. Between June 1, 2011 and August 30, 2013, ARIC conducted a fifth examination. Cognitive testing was started in 1990 and repeated in subsequent 2 exams. (https://sites.cscc.unc.edu/aric/CohortDescription)

The Coronary Artery Risk Development in Young Adults (CARDIA) Study is an on-going study examining the development and determinants of (sub-) clinical cardiovascular disease and their risk factors. It began in 1985-6 with a cohort of 5115 black and white men and women aged 18-30 years selected to balance subgroups of race, gender, education (high school or less and more than high school) and age (18-24 and 25-30) in each of 4 centers (Universities of Minnesota, Alabama at Birmingham, Northwestern and Kaiser Permanente Northern California. The DSST was first measured in the Y25 follow-up exam (2010-2011). All exams from each center have been approved the respective IRBs (https://www.cardia.dopm.uab.edu/cardia-overview/overview-more).

The Cardiovascular Health Study (CHS) is a population-based longitudinal study of coronary heart disease and stroke in adults aged 65 years and older. In 1990 5201 men and women (5201) were recruited from four communities: An additional 687 African Americans were recruited after the initial baseline survey. Eligible participants were sampled from Medicare eligibility lists in each area. Those eligible included all persons living in the household of each individual sampled from the Health Care Financing Administration (HCFA) sampling frame, who were 65 years or older at the time of examination, were noninstitutionalized, were expected to remain in the area for the next three years, were able to give informed consent and did not require a proxy respondent at baseline. Participants were followed yearly from 1988/89 to 1998. Since 1989/99, participants have been contacted every 6 months by phone, primarily to ascertain health status and for events follow-up through 2015. Cognitive testing started at baseline (https://chs-nhlbi.org/CHSOverview).

The Multi-Ethnic Study of Atherosclerosis (MESA) is a study of the characteristics of subclinical cardiovascular disease and the risk factors that predict progression to clinically overt cardiovascular disease or progression of the subclinical disease. The population-based sample of 6,814 includes asymptomatic men and women aged 45-84 from six field centers across the United States. Approximately 38 percent of the recruited participants are white, 28 percent African-American, 22 percent Hispanic, and 12 percent Asian,

predominantly of Chinese descent. Six exams have been completed since 2000. Participants are contacted every 9 to 12 months throughout the study to assess clinical morbidity and mortality. Cognitive testing started in the 5th exam (https://www.mesa-nhlbi.org/aboutMESA.aspx)./

Online Resource 2: Mean age (years) and calendar year by exam per cohort

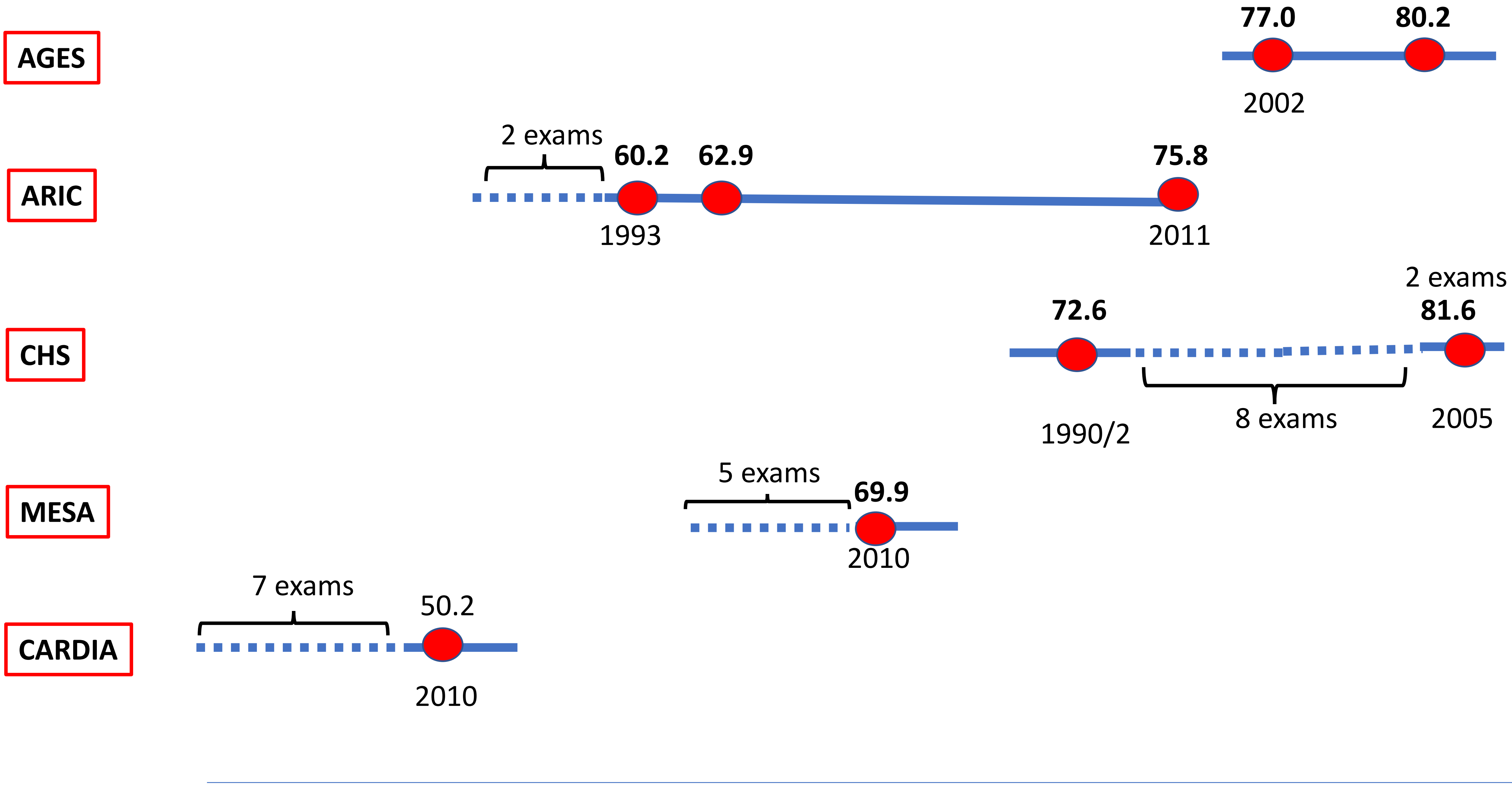

Red dots indicate exams where cardio-vascular risk factors and DSST were measured; numbers above are mean age at the exam; number below is calendar year the exam was performed.

Online Resource 3: Graphed sample size per 1-yr age bin - all cohorts combined

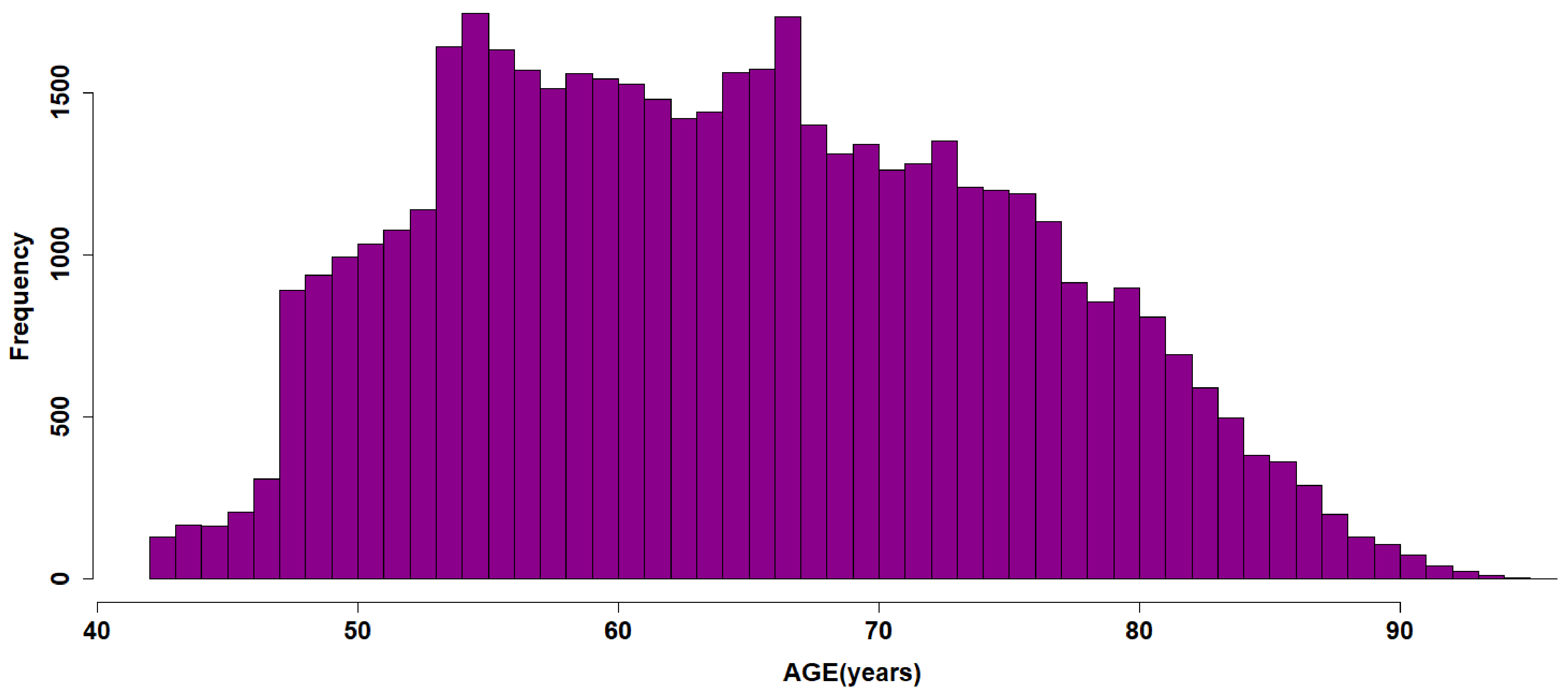

Online Resource 4: Method to harmonize risk factors and DSST across 5 cohorts in the pooled analysis

Briefly, per $cohort_{1\text{-}5}$ per risk factor ($RF_{1\text{-}5}$) we performed the following steps. In step 1, per cohort, a linear regression model estimated the predicted value of the CVRF-DSST association and the residuals (observed minus predicted values per individual). For example, in cohort 3 we generated $CVRF_{34ij}$ which stands for the prediction in cohort 3 of risk factor 4, for individual i and timepoint with corresponding residual R_{34ij}. To harmonize the scores we chose MESA as the reference. In each of the four cohorts (excluding MESA), we then calculated a predicted MESA score per risk factor, individual i and timepoint j by using as weights, the Step 1 covariate betas (per risk factor) from MESA. For example, a predicted CVRF_MESA for cohort 3, and risk factor 4, individual i and timepoint j is given by $CVRF\_MESA_{34ij} = Smoking\_value_{34ij} * \beta_{smoking\_MESA\_4} + Sex\_value_{34ij} * \beta_{male\_MESA\_4}$). This reflects the expected value of $CVRF_{34ij}$ if the individual i with timepoint j from cohort 3 and RF 4 had been in MESA but with his/her same set of covariates from cohort 3. To obtain the final harmonized value, the residual from Step 1 (i.e., R_{34ij}) was added to the predicted value from Step 2 ($CVRF\_MESA_{34ij}$) to account for the 'individual's unique deviation' relative to their own prediction in cohort 3. Thus, the harmonized score for cohort 3, RF 4, individual i and timepoint j, will be given by $H_{34ij} = CVRF\_MESA_{34ij} + R_{34ij}$. For the MESA cohort, the harmonized values are exactly the original data values from MESA.

Online Resource 5: Graphed steps to harmonize original cohort data to the MESA study

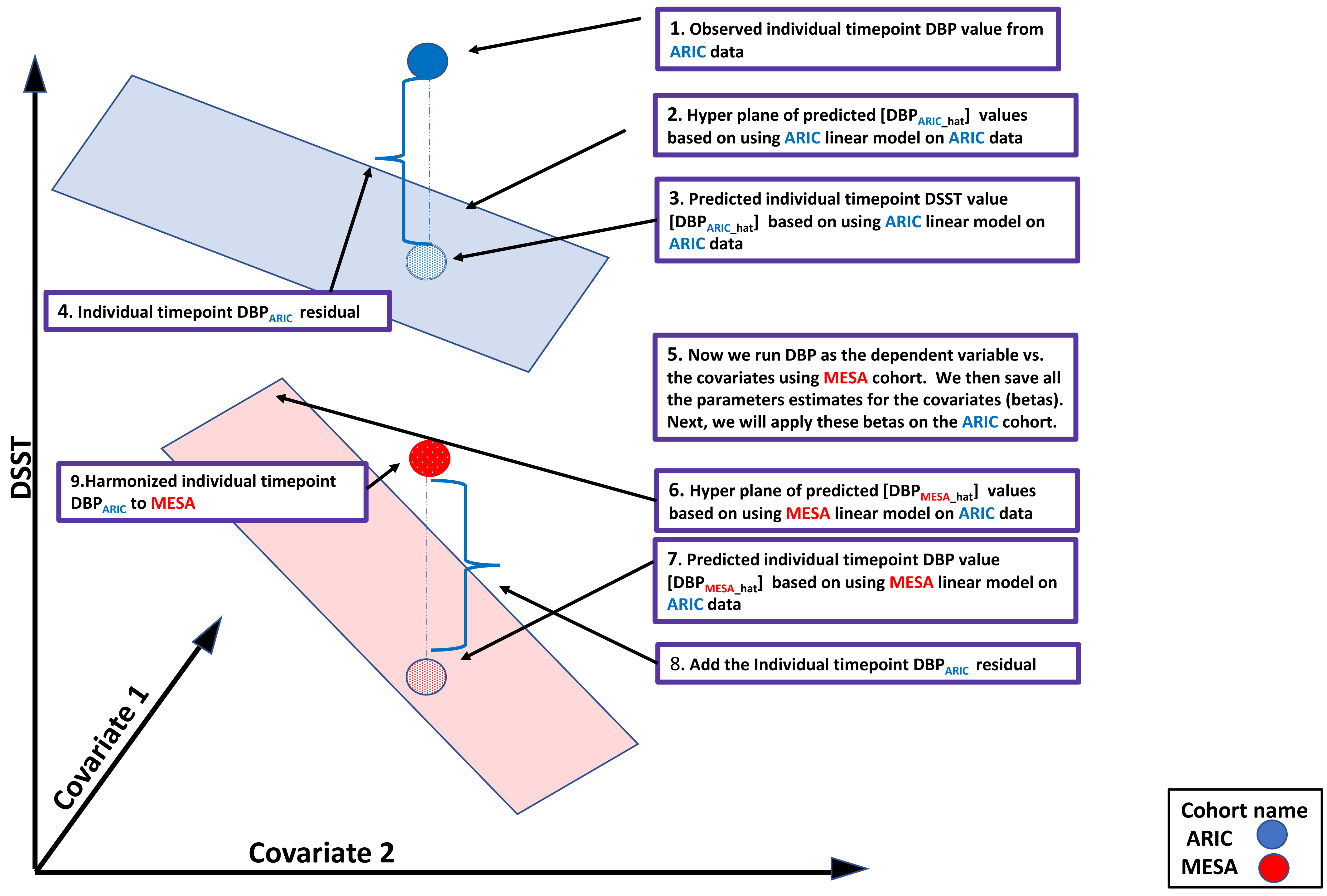

Online Resource 6a: Comparison of non-harmonized and harmonized 1-yr means of CVRF by cohort

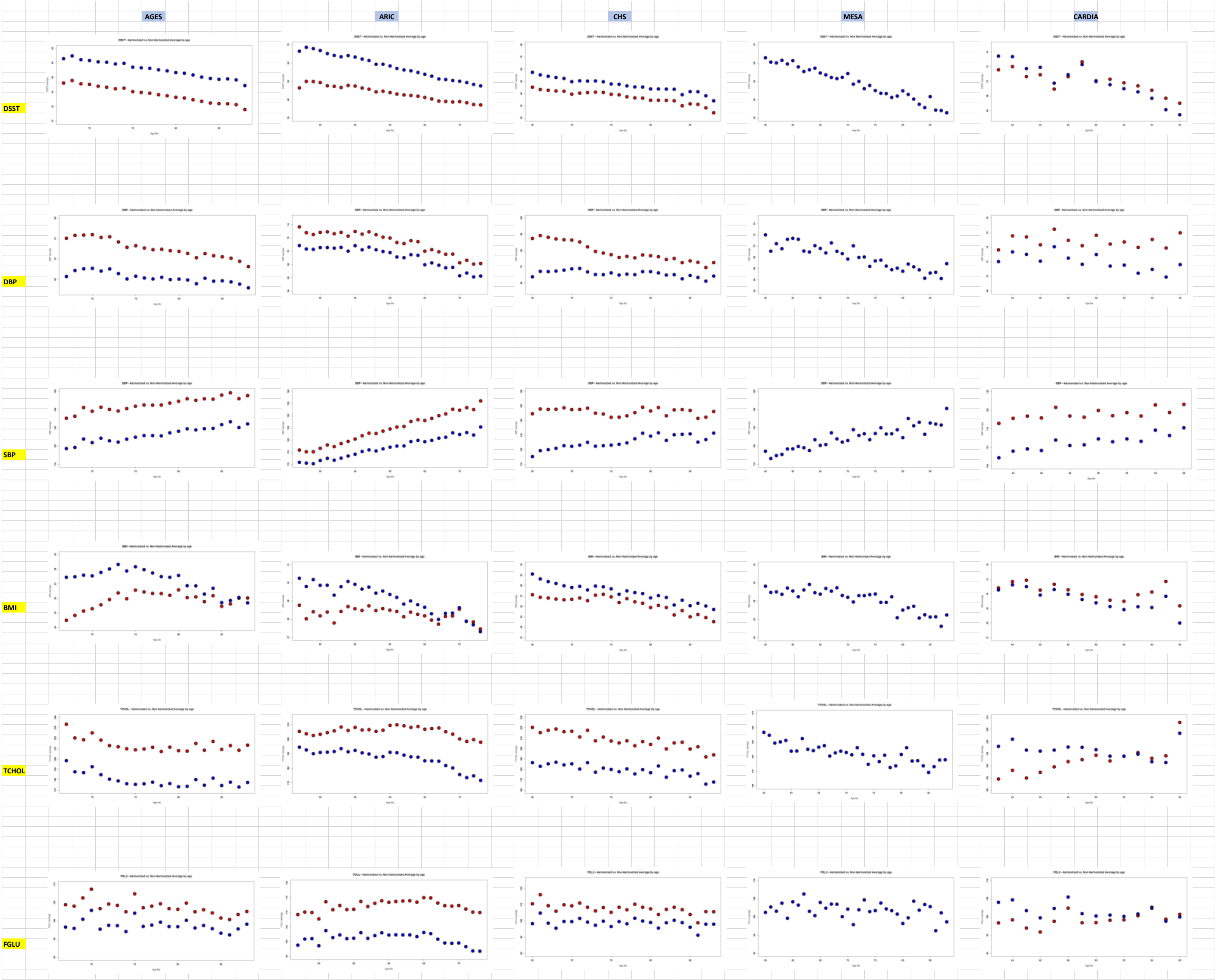

Blue dots are harmonized data and red dots and non-harmonized data

Online Resource 6b: Comparison of non-harmonized and harmonized risk factor by age by cohort

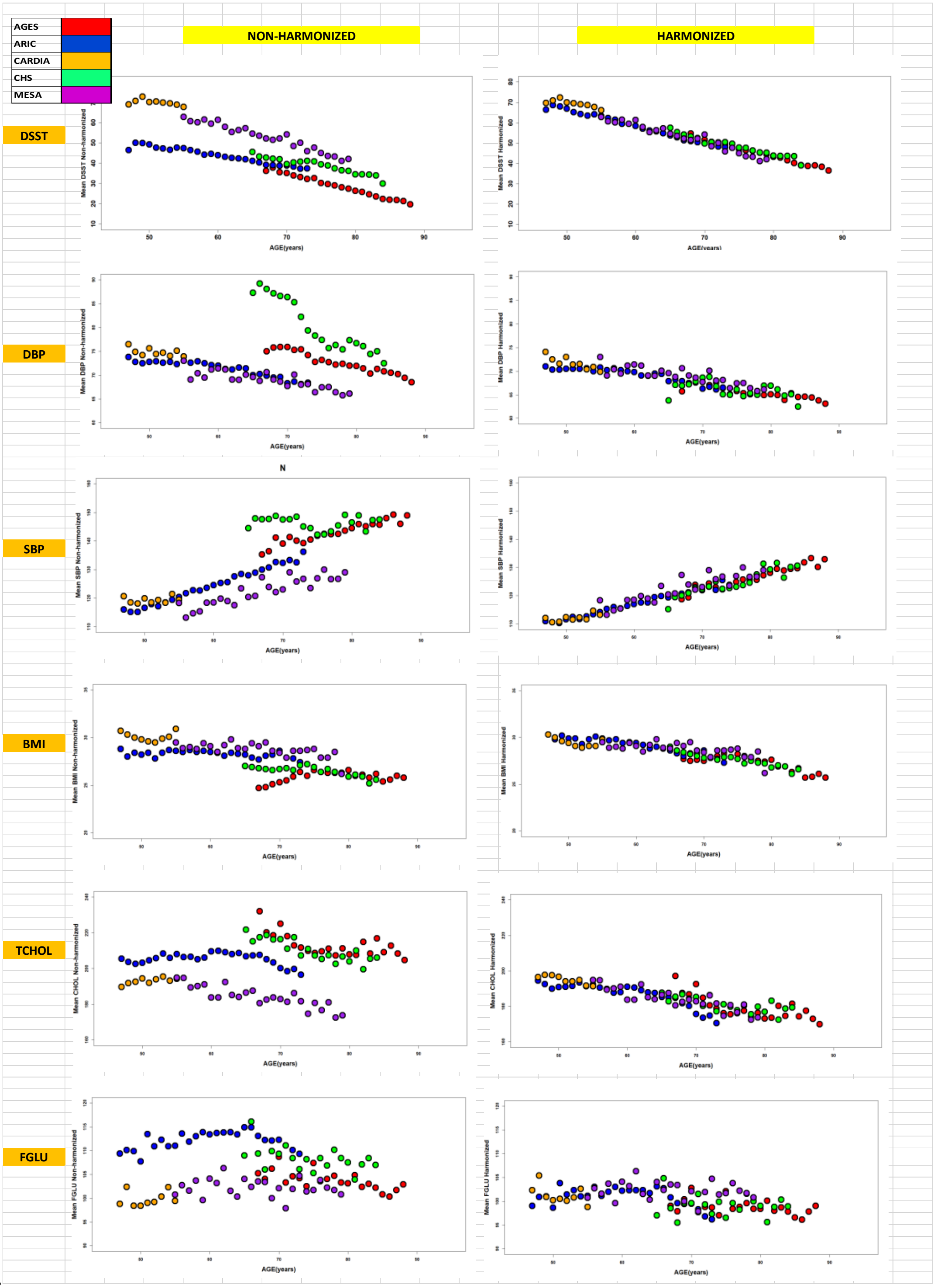

Legend: Dots are 1-yr slopes of the association between the DSST and CVRF. Red dots are from the AGES study; blue from ARIC; orange from CARDIA; CHS in green and MESA in purple.

Online Resource 7: Comparison of DSST-DBP 1-yr slope trajectories model-fit statistic without (R2_0) and with (R2_1) additional confounders (BMI, Fasting Glucose and Systolic BP).

AGES

	R2_0	R2_1	MSE_0	MSE_1
**DSST**	31.0%	31.2%	85.2	85.0
**DBP**	5.9%	7.2%	99.8	98.4
**SBP**	2.0%	2.6%	418.3	415.8

ARIC

	R2_0	R2_1	MSE_0	MSE_1
**DSST**	51.0%	51.3%	95.2	94.5
**DBP**	9.0%	11.1%	97.3	95.0
**SBP**	10.8%	14.1%	327.6	315.5

CARDIA

	R2_0	R2_1	MSE_0	MSE_1
**DSST**	26.6%	27.0%	192.0	191.1
**DBP**	9.9%	18.7%	111.7	100.8
**SBP**	11.4%	15.4%	223.3	213.1

CHS

Age < 75				
	R2_0	R2_1	MSE_0	MSE_1
**DSST**	33.8%	33.9%	84.7	84.6
**DBP**	6.6%	7.9%	101.4	100.0
**SBP**	1.8%	2.4%	428.5	426.0

Age >=75				
	R2_0	R2_1	MSE_0	MSE_1
**DSST**	29.2%	29.3%	84.3	84.2
**DBP**	5.7%	6.6%	97.3	96.4
**SBP**	2.6%	3.2%	415.9	413.5

MESA

	R2_0	R2_1	MSE_0	MSE_1
**DSST**	40.1%	41.1%	201.6	198.1
**DBP**	10.2%	12.3%	90.9	88.8
**SBP**	9.2%	12.0%	387.3	375.3

R0 model includes age, sex, smoking and education.

Online Resource 8a: Trajectories of the association of the DSST to CVRF by race.

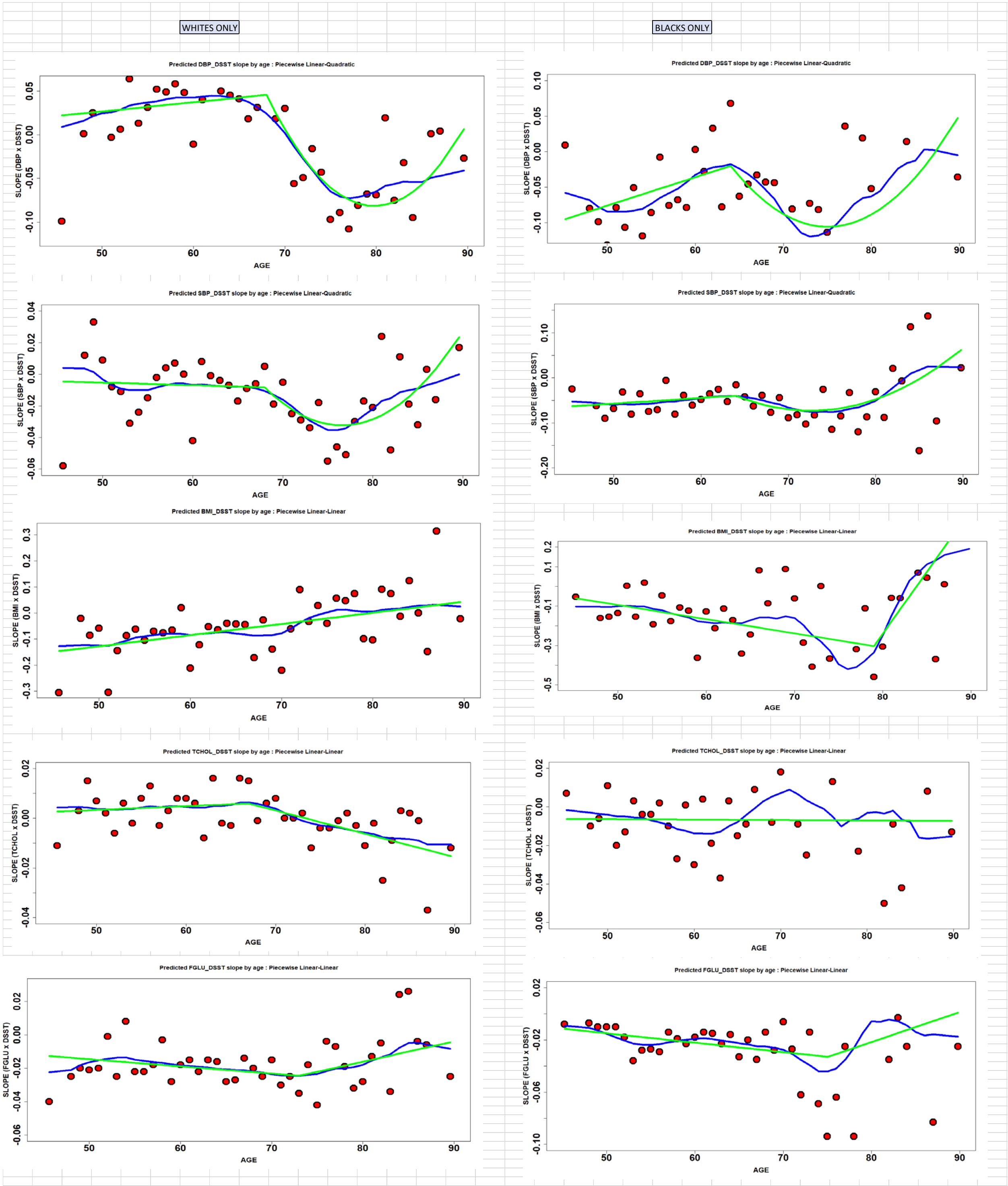

Legend: Each red dot represents the slope of the association between the DSST and cardiovascular risk factors. Trajectory lines in green are based on modeled slopes; trajectory lines in blue represent smoothed slopes.

Online Resource 8b: Trajectories of 1-yr slopes of the association of DSST to CVRF by sex

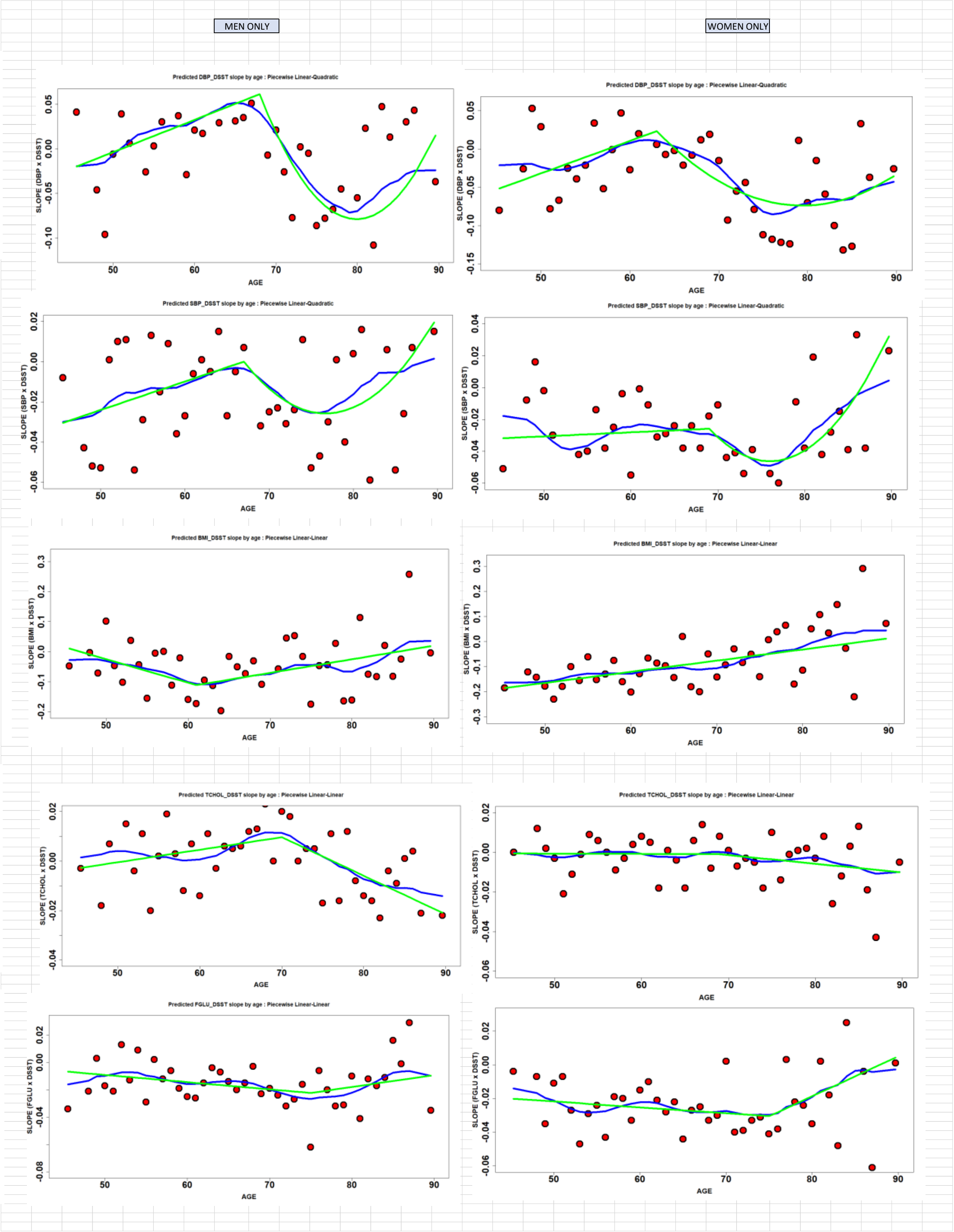

Online Resource 9: Model fit differences (R2) comparing stratified harmonized models (Race: White and Black; Sex: Men and Women) to pooled sample model (Figure 1a-e, main text)

	Standard (adjusted R-squared)				
	DBP	SBP	BMI	TCHOL	FGLU
White	65.2%	26.0%	21.2%	29.9%	6.7%
Black	8.5%	16.2%	16.9%	-2.5%	2.6%
Men	36.2%	7.6%	11.9%	14.1%	3.9%
Women	30.1%	8.9%	28.2%	-1.5%	7.4%
Pooled	58.7%	28.4%	36.8%	20.6%	9.3%

Each category (in blue) has each of the five risk factors (RF) compared with their corresponding RF in the pooled category (with all five cohorts). Highlighted in pink we have the RF in which the R2 was greater or equal to the R2 in their corresponding pooled RF.

Online Resource 10: Comparison of statistical fit for different piecewise models estimating 1-yr slope trajectories of the association between a cardiovascular risk factor and the DSST cognitive test outcome.

		Linear			piecewise linear-linear					piecewise linear-quadratic						
		slope0	int0	BIC0	acut1	slope1A	int1A	slope1B	BIC1	acut2	slope2A	int2A	QuadB	age vx	intB	BIC2
DBP	mean	-0.0021	0.12			0.0036	-0.20	-0.0046			0.0029	-0.16	0.00072	79.5	4.26	
	SD	0.00045	0.030			0.0014	0.079	0.00072			0.0010	0.06	0.00014	1.00	0.77	
	pvalue	**2.8E-06**	**4.2E-05**	-135	62	**8.4E-03**	**1.1E-02**	**3.3E-10**	-144	67	**5.3E-03**	**9.1E-03**	**2.4E-07**	**0.0E+00**	**3.2E-08**	-157
	Low	-0.0030	0.064			0.00093	-0.355	-0.0060			0.00085	-0.3	0.00045	77.5	2.8	
	High	-0.0012	0.18			0.0063	-0.046	-0.0031			0.0049	0.0	0.00099	81.5	5.8	
SBP	mean	0.0002	-0.04			-0.0005	0.01	0.0035			0.00083	-0.07	0.00029	75.4	1.57	
	SD	0.00026	0.018			0.0003	0.023	0.0011			0.00062	0.04	0.000076	1.00	0.423	
	pvalue	**4.1E-01**	**3.5E-02**	-205	77	**1.4E-01**	**7.3E-01**	**1.0E-03**	-211	67	**1.8E-01**	**6.0E-02**	**1.3E-04**	**0.0E+00**	**2.1E-04**	-214
	Low	-0.00029	-0.072			-0.0012	-0.037	0.0014			-0.00038	-0.1	0.00014	73.4	0.7	
	High	0.00071	-0.0027			0.00016	0.053	0.0057			0.0020	0.0	0.00044	77.3	2.4	
BMI	mean	0.0036	-0.32			0.0010	-0.18	0.0094			0.00088	-0.17	-0.00015	107.5	-1.74	
	SD	0.0010	0.063			0.0016	0.09	0.0029			0.0016	0.10	0.00052	336.8	3.1	
	pvalue	**2.6E-04**	**2.8E-07**	-107	70	**5.0E-01**	**6.1E-02**	**1.5E-03**	-110	70	**5.9E-01**	**8.7E-02**	**7.7E-01**	**7.5E-01**	**5.7E-01**	-106
	Low	0.0017	-0.44			-0.0020	-0.36	0.0036			-0.0023	-0.4	-0.00118	-552.7	-7.7	
	High	0.0055	-0.20			0.0041	0.008	0.015			0.0041	0.0	0.00087	767.7	4.3	
TCHOL	mean	-0.0002	0.01			0.00024	-0.01	-0.00085			0.0003	-0.01	0.00003	91.3	0.25	
	SD	0.00011	0.008			0.00023	0.014	0.00028			0.00023	0.01	0.000052	252.5	0.31	
	pvalue	**5.2E-02**	**6.3E-02**	-279	69	**2.9E-01**	**3.5E-01**	**2.2E-03**	-283	70	**2.7E-01**	**3.3E-01**	**5.2E-01**	**7.2E-01**	**4.2E-01**	-280
	Low	-0.00044	-0.0008			-0.00021	-0.041	-0.0014			-0.00020	0.0	-6.8E-05	-403.5	-0.4	
	High	1.9E-06	0.029			0.00070	0.014	-0.00030			0.00072	0.01	0.00013	586.1	0.8	
FGLU	mean	-0.0001	-0.01			-0.00035	0.00011	0.0015			-0.00021	-0.01	-0.00328	86.4	-24.46	
	SD	0.00016	0.010			0.00019	0.012	0.00078			0.00016	0.01	0.001760	4.5	12.99	
	pvalue	**5.2E-01**	**1.5E-01**	-264	75	**7.1E-02**	**9.9E-01**	**5.7E-02**	-266	83	**2.0E-01**	**4.4E-01**	**6.2E-02**	**4.2E-83**	**6.0E-02**	-264
	Low	-0.00041	-0.034			-0.00073	-0.024	-0.000043			-0.0005	-0.03	-0.006730	77.6	-49.93	
	High	0.00021	0.0052			3.0E-05	0.024	0.0030			0.0001	0.01	0.000170	95.2	1.00	

Online Resource 11: Description of the model development to estimate trajectories of 1-yr slopes describing the relationship between a cardiovascular risk factor and the DSST

1. Univariate linear model (model1)
Linear regression of CVRF-DSST 1–yr–slopes across age bins, where the predicted CVRF-DSST 1–yr–slopes for a given value of age is denoted as *T–slope*.

$$T\text{–}slope = \beta_0 + \beta_1 * \text{age}.$$

2. Piecewise models:

In these models, we defined the age of a significant change in *T–slope* as *agecut*, where flag variables F1 and F2 are defined as:

$$F_1 = 1 \text{ if age} \leq agecut \text{ and F1} = 0 \text{ otherwise}$$

$$F_2 = 1 \text{ if age} > agecut \text{ and F2} = 0 \text{ otherwise}$$

where in model 2a (see below) slope parameters that are estimated for 'piece 1' are denoted as *T–slope1*, and for piece 2 as *T–slope2*. Similarly, we will refer to the intercept for piece 1 as in stage1. Given the fact that the line from the regression in piece 1 is connected to the line from the regression in piece 2 (at age = *agecut*), there is one less degrees of freedom such that the intercept in part 2 is a function of *T-slope2* and *agecut* so it does not need to be estimated by regression but can be easily calculated if needed. The age at which the strongest change in direction occurs (*agecut*) was selected based on the piecewise model with the lowest Bayesian Information Criteria (BIC) (across all values of *agecut* in the interval 55 to 85 y) obtained from running a different model for each *agecut*.

2a. *Piecewise linear-linear model*. A constraint is set so the regression line in piece 2 is a continuation of the regression line in piece 1; so the predicted CVRF-DSST 1–yr–slope should be exactly the same for both pieces at the point where age is equal to *agecut*.

Next, let TX be defined as F1*(age – *agecut*) + *agecut*. Thus, our model is defined as:

$$T\text{–}slope = \beta_0 + \beta_1 * \text{TX} + \beta_2 * \text{age}.$$

Here, the estimate for the *T–slope1* in piece 1, is equal to $\beta_1 + \beta_2$ whereas the intercept estimate is equal to β_0. For piece 2, the *T–slope2* estimate is β_2. The intercept for *T–slope2* is equal to $\beta_0 + (\beta_1 * agecut)$, although we do not use the *T–slope2* intercept in this study.

2b. Piecewise linear/–quadratic model. In piece 1 we modeled a univariate linear regression just as in model 2a. For piece 2, we model CVRF-DSST 1yr–slopes as a quadratic function of age that is connected to the regression line in piece 1. In addition, although we will model the quadratic function in the standard form, we are also interested in the vertex, that is, the estimate of the age of relative minimum, which we

will use in our analysis. Just as for model 2a, different age cutoffs were tested for different values of age between 55 and 85 and the *agecut* used in the model with smallest BIC was selected. The parameters of the model are defined as $F_1 = 1$ if age <= *agecut* and $F_1 = 0$; otherwise, $F_2 = 1$ if age > *agecut* and $F_2 = 0$, and in addition, a constraint is set so the quadratic function in piece 2 is a continuation of the line in piece 1 at age = *agecut*.

$$\text{Let } TX_1 = F1 * (\text{age} - \textit{agecut})$$

$$TX_2 = (\text{age}^2 - \textit{agecut}^2)*(1-F1)$$

$$TX_3 = (\text{age} - \textit{agecut}).$$

Thus, our model is defined as:

$$\textit{T–slope} = \beta_0 + \beta_1 * TX_1 + \beta_2 * TX_2 + \beta_3 * TX_3$$

Here, the estimate for (the age coefficient) in piece 1, is equal to $\beta_1 + \beta_3$, whereas the intercept for piece 1 is equal to $\beta_0 - \textit{agecut}*(\beta_1 + \beta_3)$. In piece 2, the linear coefficient estimate (the age coefficient) is β_3, the quadratic coefficient estimate (the age^2 coefficient) for piece 2 is β_2 and the estimate for the age of relative minimum in piece 2 is equal to $-\beta_3/(2*\beta_2)$ and the estimate for the intercept for piece 2 (though not used in this study) is $\beta_0 - (\beta_2*\textit{agecut}^2) - (\beta_3*\textit{agecut})$.

3. Description of calculating the 95% CI

For each RF, we start by using the same agecut from the corresponding model (i.e. linear-quadratic). Next, we perform 1000 iterations (bootstraps) where the parameter estimates of a predictor variable are estimated for each iteration. We save the parameter estimates for each iteration into a vector (1000 values). Finally, we sort the vector and select the values that correspond to the 2.5%-ile and 97.5%-ile (positions 25 and 975). These values correspond to a 95% CI.

Online resource 12: Description of the smoothing algorithm applied to the trajectories of 1-yr slopes shown in Figures 1a-e (main text)

1. Assume that we have 50 timepoints with weights w_1, w_2, …,w_{50}
2. In this study, each weight is the inverse of the square of the standard error per bin.
3. Let the vector (1,2,3,…,6,5,4,…,2,1) be a vector of moving average weights, such that the value '6' is aligned with a timepoint xi.
4. The 11pt. triangle moving average, centered on timepoint x_{15} is given by:

$$(x_{10}*w_{10}*1 + x_{11}*w_{11}*2 + \ldots + x_{15}*w_{15}*6 + x_{16}*w_{16}*5 + \ldots + x_{20}*w_{20}*1) \;/$$

$$w_{10}*1 + w_{11}*2 + \ldots + w_{15}*6 + w_{16}*5 + \ldots + w_{20}*1)$$

5. If a point is too close to one extreme (i.e., x_3) a truncated 11pt.triangle average centered on x_3 is given by:

$$(x_1*w_1*4 + x_2*w_2*5 + \ldots + x_3*w_3*6 + x_4*w_4*5 + \ldots + x_8*w_8*1) \;/$$

$$(w_1*4 + w_2*5 + \ldots + w_3*6 + w_4*5 + \ldots + w_8*1)$$

so, in this case instead of a moving average of 11pt it will be a moving average of 8 points due to truncation.

In summary, since the value '6' is centered on x_i, then for any $i \leq 5$ or for an $i > n-5$, where n is the total number of time points, we will have a truncated moving average.

Online Resource 13: Table: Cross cohort harmonized and non-harmonized mean and standard deviation of risk factors and DSST cognitive score

	DSST	SBP	DBP	BMI	TCHOL	FLGU
**MEAN**	42.8	130.2	72.9	27.8	204.9	108.7
**MEAN-H**	54.9	119.8	68.3	28.6	185.3	100.7

	DSST	SBP	DBP	BMI	TCHOL	FLGU
**SD**	17.0	22.0	11.4	5.4	40.5	35.7
**SD-H**	15.8	20.0	10.9	5.3	40.5	35.1

Mean-H = Harmonized Mean; SD= Standard Deviation; SD_H = Harmonized standard deviation;
DSST= Digit Symbol Substitution Test; DBP=Diastolic Blood pressure; SBP= Systolic Blood pressure; BMI=Body mass index;
TCHOL=Total cholesterol; FGLU= Fasting glucose.

Online Resource 14: Trajectories of harmonized 1-yr slopes of the association between the DSST and cardiovascular risk factors by cohort.

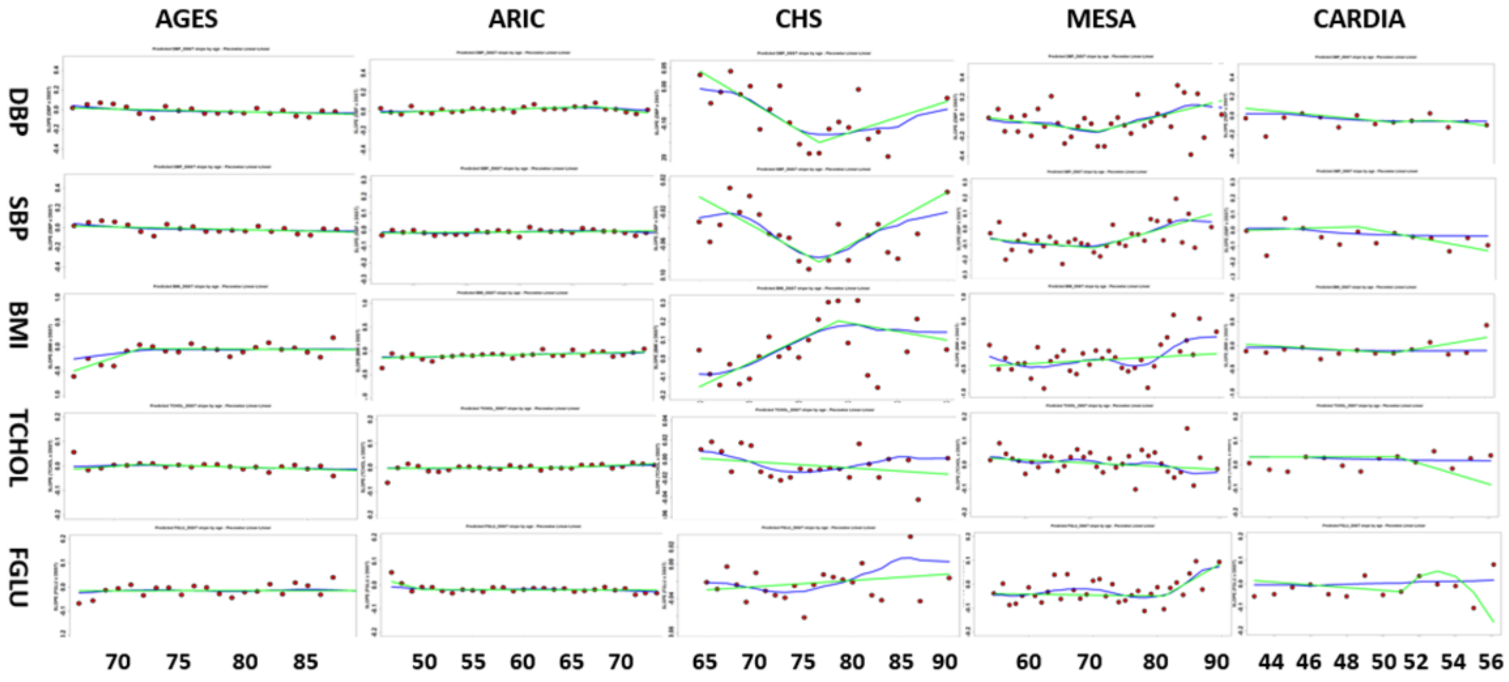

Legend: Each red dot represents the slope of the association between the DSST and cardiovascular risk factors. Trajectory lines in green and based on modeled (estimated) slopes; trajectory lines in blue represent smoothed slopes
